# Severe COVID-19 infection in a patient with a blastic transformation of a chronic myeloid leukemia and severe treatment-induced immunosuppression: a case report

**DOI:** 10.11604/pamj.supp.2020.37.1.25501

**Published:** 2020-11-11

**Authors:** Louardi Mounir, Simou Mehdi, Fahmaoui Kawtar, Mansour Akram, Et-tahir Youness, Tabat Meryem, Joutey Tahiri Othmane, Elkhaouri Imane, Ezzouine Hanane, Charra Boubakar, Camara Marieme, Lamchahab Mouna, Harrach Asmaa, Quessar Asmaa

**Affiliations:** 1 Medical Intensive Care Unit, Ibn Rochd University Hospital of Casablanca, Hassan II University of Casablanca, Casablanca, Morocco,; 2 Faculty of Medicine and Pharmacy, Hassan II University of Casablanca, Casablanca, Morocco,; 3 Hematology Department, Ibn Rochd University Hospital of Casablanca, Hassan II University of Casablanca, Casablanca, Morocco

**Keywords:** Leukaemia, chemotherapy, COVID-19

## Abstract

The severe acute respiratory syndrome coronavirus 2 (SARS-CoV-2) has rapidly spread across the globe, leading to the declaration of a pandemic. While most present mild symptoms, it appears as though nearly 20% of confirmed patients develop significant complications. At this time of uncertainty, we are struggling to provide appropriate care to hematological cancer patients. We need to weigh the risks and benefits of giving cancer treatment against the odds of infecting them with COVID-19. As hematological cancer patients are immunocompromised and there are high chances of exposure during hospital visits, they can get infected and outcome can be fatal. So in this case report, we intend to discuss the possible impact of the current COVID-19 pandemic on patients with acute leukaemia in terms of diagnosis, chemotherapy, and prophylactic measures.

## Introduction

The ongoing COVID-19 pandemic caused by severe acute respiratory syndrome coronavirus 2 is a global public health crisis. It has now infected over fourteen million people across the globe, taking the lives of over six hundred thousand people, and leaving many with irreparable damage. The most common clinical symptoms of COVID-19 range from fever, cough, fatigue, dyspnea, and in some cases nausea, diarrhea and vomiting. However, symptom reporting is unreliable in subjects with hematological cancers who may have other reasons for developing fever, cough etc, especially those receiving anti-cancer therapy. Moreover, they are more likely to get infected and at large risk of COVID-19 serious events due to their immunosuppressed state, related to the leukaemia itself or the chemotherapy. In this case report, we intend to discuss the possible impact of the current COVID-19 pandemic on patients with acute leukaemia in terms of diagnosis, chemotherapy, and prophylactic measures.

## Patient and observation

**Figure 1:**
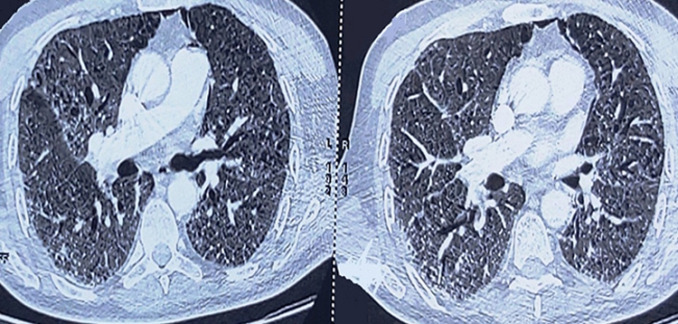
a chest CT-scan showing bilateral condensation and ground glass opacities in favor of an infectious pneumonia

A 28 years old female patient, with a six months history of a chronic myeloid leukaemia (CML) treated by tyrosine kinase inhibitors leading to clinical and biological improvement, was admitted in the hematology ward on the 28^th^ of June, due to a 4 days history of diarrhea and fever up to 40°C. White blood cell count showed a leukocytosis of 100000 el/mm^3^, with a blast percentage of 90%. A bone marrow aspiration and immunophenotyping were realized and revealed a blastic transformation of her CML into an acute myeloid leukaemia (AML) M4 subtype. A chest X-ray was performed as part of the septic workup showing a right lower lobe pneumonia, followed by a chest CT scan (Figure 1) showing bilateral condensation and ground glass opacities in favor of an infectious pneumonia associated with a small amount of fluid in the right pleural cavity. The patient received antibiotics and a first cure of chemotherapy.

As routine evaluation in all patients with hematological malignancies, the patient was tested for SARS-CoV-2. Reverse transcriptase polymerase chain reaction (RT-PCR) from nasal and pharyngeal swab came back positive. Following a deterioration of her general health status, the patient was transferred to the ICU on the 2^nd^ of July for SARS-CoV-2 pneumonia. Upon admission, the medical examination revealed a conscious patient (GCS 15/15), respiratory rate at 46 cycle per minute, oxygen saturation (SpO2) of 70% at room air and 95% upon wearing a non-rebreather face mask (15 L/min), blood pressure of 100/50 mmHg, pulse of 110 bpm and a temperature of 40°C. Complete blood count showed a leukocytosis, white blood cells of 158140 el/mm^3^, neutrophils of 1580 el/mm^3^, lymphocyte of 6330 el/mm^3^, anemia with a hemoglobin at 6.2 g/dl, thrombocytopenia with platelets of 32000. Blood hemostasis analysis showed a biological disseminated intravascular coagulation (DIC), prothrombin time at 48% and partial thromboplastin time at 39.7 sec, fibrinogen at 2.58, D-dimers at 6700 mg/l. Biochemical profile showed an hypokalemia of 1.5 mmol/l, CRP of 106, PCT of 1.68, ferritin at 2000 ng/l and troponins at 70 ng/l, without any tumour lysis syndrome was absent: uric acid< 5, LDH at 3271 UI/l, potassium at 1.5 mmol/l, phosphorus at 27 mg/l, calcium at 85 mg/l. A pretherapeutic EKG showed a sinus tachycardia at 100 bpm, no repolarization trouble and a normal QTc at 464 ms. The echocardiography showed: for the left ventricle: normal in size with normal wall thickness and systolic function, an ejection fraction at 50%, without neither dyskinesia nor thrombosis. For the right ventricle: no valvular leakage or stenosis, a right ventricle systolic function associated with a concentric left ventricular hypertrophy. Both aorta and the inferior vena cava are normal.

**Figure 2:**
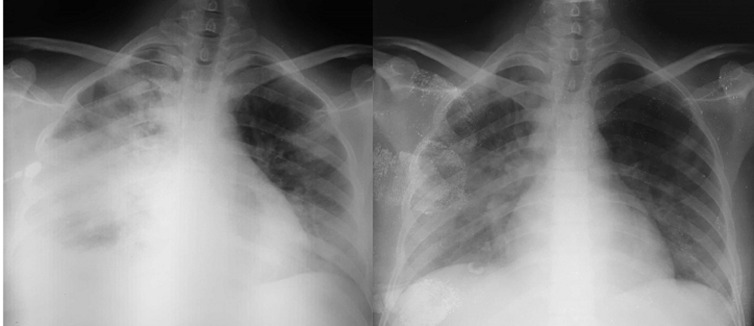
a chest X-ray on admission (on the left), compared to a chest X-ray on day 9 (on the right), showing a radiological improvement after nine days of treatment

An infectious workup, consisting of urine cytobacteriological examination, stool tests, a PCR based detection of mycobacterium tuberculosis, blood cultures and aspergillus serology all came back negative. Therapeutic management included oxygen therapy, non-invasive ventilation at 50% FIO2 (PEP at 5 and IA at 12). Medical treatment associated hydroxychloroquine 200 mg 3 times a day, azythromycin 500mg the first day then 250mg per day, methylprednisone at 80mg a day for 7 days and curative anticoagulation treatment including enoxaparin 100 UI/kg (1mg/kg) twice a day. On day 3, the patient was eupneic (SpO_2_of 98%) wearing nasal cannula at 3L/min, she no longer needed sessions of non-invasive ventilation. One week later, the patient had a favorable clinical and radiological (Figure 2) outcome and was declared cured of the SARS-CoV-2 infection following a negative PCR test, she was transferred back to the hematology ward for further treatment of her acute myeloid leukaemia.

## Discussion

Multiple observations indicate a higher risk for severe, potentially life-threatening illness related to COVID-19 in older people and patients with underlying conditions, compared to the general population [1]. This observation presents a particular concern for patients with myeloid neoplasm, including acute myeloid leukaemia. Early data from the Chinese National Database Repository suggest an over-representation of patients with cancer in the COVID-19 cohort. COVID-19-related case fatality rates are estimated to be 5-6% in those with cancer. Moreover, patients with cancer are at a 3-5 times higher risk of severe COVID-19 compared with the general population. Additionally, case fatality rates of up to 37% have been reported in patients with hematological malignancies and COVID-19 [2]. However, the exact incidence of COVID-19 in patients with cancer in general, ad in patients with leukaemia in particular, is unclear. Considering the challenges faced by the oncology community in managing adult patients with myeloid neoplasm, during this pandemic, major medical societies, including the American Society of Hematology, have released resources to help providers care for patients with hematological malignancies.

Regarding the diagnosis, patients with acute leukemia are at large risk of missed or delayed detection of the COVID-19 infection. In 50-75% of patients with AML, symptoms may get confused with the cancer's symptoms [3]. For this reason, an RT-PCR testing for SARS-CoV-2 infection should be part of the routine evaluation in all patients with hematological cancers. In addition to diagnosis delay, most patients may suffer from postponed chemotherapy, due to a shortage of isolation beds and blood products or the wish to avoid immunosuppressive treatments. Delay in chemotherapy initiation may negatively affect prognosis, particularly in young (<60 years-old) patients. Indeed, they could progress to high-risk disease following the acquisition of additional genetic anomalies, and hyperleukocytosis. More treatments may then be needed to achieve a deep complete remission before allogeneic stem cell transplantation [3]. Moreover, patients may suffer from delay or deferral in hematopoietic stem cell transplantation and blood products shortage [3]. As for the treatment, the health-care provider must carefully balance the risks and the benefits when taking a decision. Because the administration of intensive chemotherapy amid an active pandemic can place patients at a high risk of contracting a severe SARS-CoV-2 infection, with potentially fatal consequences, and might lead to intensive care unit [2]. And so, one should consider delaying treatment if possible. Test all patients before initiating treatment: If the test is positive, consider delaying treatment by 10-14 days. If the test is negative, repeat the test after 24 hours if there is high clinical suspicion (RT-PCR has a positive predictive value of 70%). If the patient is positive for SARS-CoV-2 and initiation of treatment is urgently needed, consider treatment with close monitoring for any evolving and related COVID-19 symptoms [2].

As for the prophylactic measures, patients with recent or ongoing treatment for leukaemia are to be protected from COVID-19 by self-isolation, respecting basic hygiene recommendations and having up-to date, vaccination status, notably against *Streptococcus pneumoniae* [3]. Coming back to our patient, she had a recent blastic transformation of a chronic myeloid leukaemia treated by a first cure of chemotherapy, which is believed to be the cause of the patient´s deterioration. When the patient was tested positive for a SARS-CoV-2 infection, the decision was to hold up on chemotherapy, until the viral infection is managed, and the patient´s transferred back to the hematology ward. Our patient had a rather rapid improvement, although she received the first cure of chemotherapy. However, not all hematological cancer patients had the same positive outcome. They are still at increased risk of developing severe or lethal COVID-19 complications. That´s why they require timely diagnosis, evaluation, treatment and follow-up.

## Conclusion

The COVID-19 pandemic has severely strained the oncology community and the delivery of optimal care for patients with acute leukaemia and associated myeloid neoplasm. The need to treat patients with potentially lifesaving, intensive chemotherapy presents an enormous challenge. As it puts an immunocompromised population at even greater risk for contracting severe COVID-19. Setting early goals of care and discussing code status for all patients with hematological malignancies is imperative.
